# Nonresolving pleural effusion in an elderly woman: A case report

**DOI:** 10.4103/1817-1737.69118

**Published:** 2010

**Authors:** R. Garg, R. Sodhi, P. Jabeed, A. Rastogi

**Affiliations:** *Department of Pulmonary Medicine, Chhatrapati Sahuji Maharaj Medical University, (erstwhile King George’s Medical University) Lucknow, Uttar Pradesh, India*; 1 *1Department of Pathology, Chandra Dental College, Lucknow, Uttar Pradesh, India*

A 70-year-old nondiabetic and nonsmoker woman from eastern district of Uttar Pradesh, India, was referred to our department as a case of right-sided pleural effusion cause? Tubercular. She was on antitubercular treatment (ATT) for the past one and a half month with no response in her complaints of fever, breathlessness, dry cough, and right-sided chest pain. At the time of presentation, she had lowgrade continuous fever, breathlessness, and chest pain, which was in the lower part of the chest on the right side and increased on deep inspiration and coughing. On physical examination, she had reduced chest expansion, stony dull percussion, and reduced breath sounds on the right side. The rest of the examination was not contributory. Her repeat chest radiograph revealed right-sided pleural effusion [Figure 1]. She was investigated on the lines of nonresolving pleural effusion keeping the differential diagnosis of malignancy, collagen vascular disease, and tuberculosis in mind. Her routine blood investigations and biochemistry were within normal limits.

**Figure 1:**
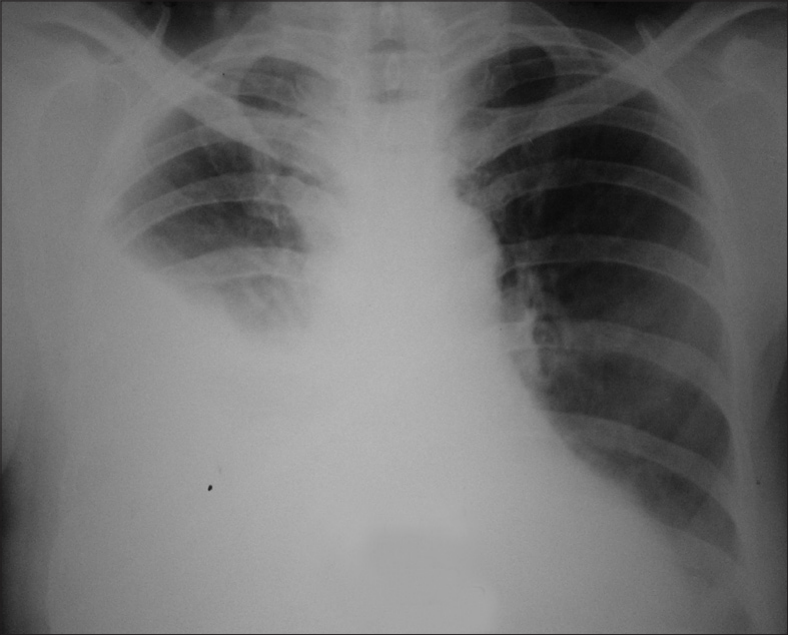
Chest radiograph at the time of presentation

The Mantoux test showed no induration. Three sputum specimens for acid fast bacilli were negative on direct smear examination. Antinuclear antibody and Antidouble stranded DNA antibody were not detected. Diagnostic thoracentesis revealed straw colored fluid. It was exudative (protein 4.2 g/dL and serum protein 7.0 g/dL) in nature with cytology showing 240 cells/mm^3^ with lymphocytes 90%, eosinophils 05%, and neutrophils 05%. Adenosine deaminase of fluid had the value of 35.5 U/L (normal < 40 U/L). No acid fast bacilli or bacteria were seen on Ziehl–Neelsen and Gram stain, respectively. Polymerase chain reaction of fluid was negative for nontubercular mycobacterium and *Mycobacterium tuberculosis*. The pleural fluid was negative for malignant cells on four different occasions. During the examination of the fluid in the Neubauer chamber for the leukocyte count, our microbiologist noticed a single parasitic organism [Figure 2].

**Figure 2:**
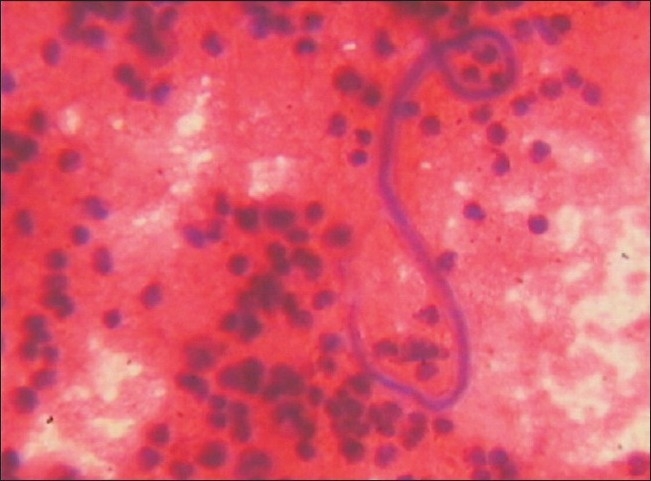
Microfilaria seen in pleural fluid cytology

## What is Your Diagnosis?

## Answer

### Right-sided pleural effusion—Cause filariasis

Filariasis is a major public health problem in India with heavily endemic areas being Uttar Pradesh, Bihar, Andhra Pradesh, Orissa, Tamil Nadu, Gujarat, and Kerala. *Wuchereria bancrofti* is the most widespread of filarial organisms infecting humans. The major clinical presentations include fever, asymptomatic microfilariaemia, lymphatic obstruction, and tropical pulmonary eosinophilia.[1] Acute manifestations are fever, adenolymphangitis, funiculitis, epididymitis, and orchitis. Lymphedema, hydrocele, elephantiasis, and chyluria are features of chronic filariasis.[2] Tropical pulmonary eosinophilia is a form of occult filariasis causing interstitial lung disease due to immunologic hypersensitivity to filarial antigen. The adult worm resides in lymphatic vessels, whereas microfilariae, the larval forms, circulate in the peripheral blood.[3] Microfilariae probably appear in tissue fluids and exfoliated surface material due to lymphatic or vascular obstruction. Diagnosis is made on demonstrating microfilaria in blood samples and body fluids.

The development of pleural effusion is a rare manifestation of filariasis and such effusions are usually exudative in nature.[3] It may be due to lymphangitis, resulting in incomplete obstruction of lymphatics. Some others have also reported about pleural effusions due to filariasis.[2,4–7] In one case, microfilariae of *Mansonella perstans*[2] were detected, whereas in others, *W. bancrofti* were present. Four of these cases were from nonendemic areas.[2] Three cases had tropical pulmonary eosinophilia, whereas one did not have peripheral eosinophilia.[4] In another case, filarial larvae were detected on pleural biopsy.[2] Four of these showed a left-sided pleural effusion.[2,6] The fluid was an exudate without eosinophilia. Reasons for exclusive left-sided effusion are not known. Only one case had tropical pulmonary eosinophilia with a bilateral transudative effusion.[5]

In India, where filariasis is endemic and the commonest cause of pleural effusion is tuberculosis, the coexistence of filariasis with pleural effusion is thought to be coincidental rather than etiologic. In our case, to rule out incidental parasitic occurrence, we made several wet films from the sediments of the fluid, which revealed numerous motile larvae. Smears showed microfilariae of *W. bancrofti* at four different occasions based on the morphology.

This case highlights the need for continued clinical vigilance when a diagnosed case of exudative pleural effusion does not behave in a manner it should on receiving ATT in an area with high prevalence of tuberculosis. Ruling out malignancy is also of prime importance. On ruling out malignancy, other causes, such as parasitic, collagen vascular disease, pancreatitis, or sarcoidosis, should be considered and a full microbiological workup should be pursued. Communication with microbiology staff is essential in the diagnostic process, which results in prompt institution of appropriate chemotherapy.

**Figure 3:**
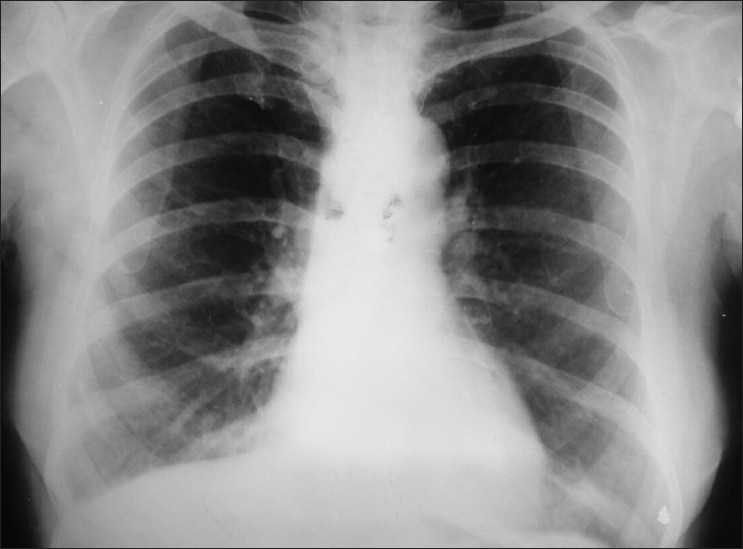
Chest radiograph after 12 months

Our patient was started on diethyl carbamazine and she responded excellently. She was given treatment for 3 months and followed-up for 1 year with no evidence of refilling or recurrent effusion [Figure 3]. The presence of microfilariae in the pleural fluid and the successful response to treatment with diethylcarbamazine is strong evidence of filarial etiology of pleural effusion.
